# Crystal structure of bis­(tri­ethano­lamine-κ^3^ *N*,*O*,*O*′)nickel(II) bis­(3-hy­droxy­benzoate) tetra­hydrate

**DOI:** 10.1107/S2056989016005521

**Published:** 2016-04-08

**Authors:** Aziz B. Ibragimov

**Affiliations:** a Institute of General and Inorganic Chemistry of Uzbekistan Academy of Sciences, M.Ulugbek Str, 77a, Tashkent 100170, Uzbekistan

**Keywords:** crystal structure, 3-hy­droxy­benzoic acid, tri­ethano­lamine, hydrogen bonding

## Abstract

The reaction of 3-hy­droxy­benzoic (*m*-hy­droxy­benzoic) acid (MHBA), tri­ethano­lamine (TEA) and Ni(NO_3_)_2_ in aqueous solution led to formation of the hydrated title salt, [Ni(C_6_H_15_NO_3_)_2_](C_7_H_5_O_3_)·4H_2_O. In the complex cation, the Ni^2+^ ion is located on an inversion centre. Two symmetry-related TEA ligands occupy all coordination sites in an *N*,*O*,*O*′-tridentate coordination, leading to a slightly distorted NiN_2_O_4_ octa­hedron. Two ethanol groups of each TEA ligand form two five-membered chelate rings around Ni^2+^, while the third ethanol group does not coordinate to the metal atom. Two MHBA^−^ anions in the benzoate form are situated in the outer coordination sphere for charge compensation. An intricate network of hydrogen bonds between the free and coordinating hy­droxy groups of the TEA ligands, the O atoms of the MHBA^−^ anions and the water mol­ecules leads to the formation of a two-dimensional structure extending parallel to (010).

## Abstract 2

In the mol­ecular cation of the title compound, the Ni^II^ ion is located on an inversion centre and is coordinated by two tridentate tri­ethano­lamine ligands. Two 3-hy­droxy­benzoate counter-anions and four lattice water mol­ecules give rise to the formation on an intricate system of hydrogen bonds.

## Chemical context   

Tri­ethano­lamine (TEA) is a substance with relatively low anti­microbial (Zardini *et al.*, 2014 ▸) and plant-growth-stimulating (Loginov *et al.*, 2012 ▸) activities. However, it is a well-known compound owing to technical applications as a curing agent for ep­oxy and rubber polymers, adhesives and anti­static agents, and as a corrosion inhibitor in metal-cutting (Ashton Acton, 2013 ▸). The inter­action of metal ions with TEA can result in the formation of complexes in which it demonstrates monodentate (Kumar *et al.*, 2014 ▸), bidentate (Long *et al.*, 2004 ▸), tridentate (Mirskova *et al.*, 2013 ▸; Haukka *et al.*, 2005 ▸) or tetra­dentate (Zaitsev *et al.*, 2014 ▸; Langley *et al.*, 2011 ▸) binding modes. TEA ligands are also able to inter­act as bridging ligands between two metal cations (Sharma *et al.*, 2014 ▸) or as bridging ligands to form one-dimensional polymeric structures (Custelcean & Jackson, 1998 ▸). Moreover, there are metal complexes in which TEA mol­ecules are non-coordinating and are consequently situated outside the actual coordination spheres (Ilyukhin *et al.*, 2013 ▸; Manos *et al.*, 2012 ▸).
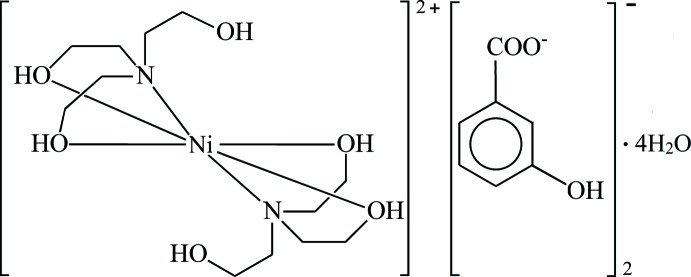


In contrast to the other two biologically active isomers of hy­droxy­benzoic acid, namely *o*-hy­droxy­benzoic (salicylic) and *p*-hy­droxy­benzoic (paraben) acid, *m*-hy­droxy­benzoic acid (MHBA) has no specific biological action. Nevertheless, MHBA is a component of castoreum, the exudate from the castor sacs of the mature North American beaver, used in perfumery and folk medicine (Müller-Schwarze & Houlihan, 1991 ▸). Most metal complexes of MHBA are in their mixed-ligand form in which mono- (Ma *et al.*, 2013 ▸; Köse *et al.*, 2012 ▸) or bidentate (Thompson *et al.*, 2015 ▸; Zaman *et al.*, 2012 ▸) coordination through the carb­oxy­lic oxygen atoms take place. The latter coordination mode may give rise to the generation of polymeric metal complexes (Koizumi *et al.*, 1984 ▸; Koziol *et al.*, 1990 ▸). There are also structures in which MHBA mol­ecules are non-coordinating (Zaman *et al.*, 2013 ▸) or simultaneously coordinating and non-coordinating (Li *et al.*, 2008 ▸).

To the best of our knowledge, metal complexes on the basis of MHBA and ethano­lamines have not yet been obtained and structurally characterized. Here, the synthesis and structure of [Ni(C_6_H_15_NO_3_)_2_](C_7_H_5_O_3_)_2_·4H_2_O is reported.

## Structural commentary   

**Figure 1:**
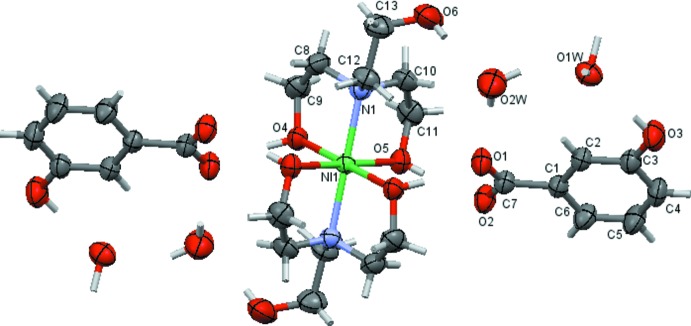
The mol­ecular entities in the title structure, with displacement ellipsoids drawn at the 50% probability level. The parts of the asymmetric unit are identified by labelled atoms; all other atoms are generated by the symmetry operation (−*x* + 1, −*y*, −*z* + 1).

The asymmetric unit of the title compound contains one half of the complex nickel(II) cation (the other part being completed by inversion symmetry), one MHBA^−^ counter-anion and two water mol­ecules (Fig. 1 ▸). Two symmetry-related TEA ligand mol­ecules coordinate in a *N*,*O*,*O*′-tridentate binding mode to the metal cation, giving rise to a slightly distorted octa­hedral NiN_2_O_4_ coordination environment. One hydroxyl group of each ethanol substituent is not involved in the coordination and is directed away from the coordination centre. As a result of symmetry requirements, the nitro­gen atoms are in *trans*-positions of the coordination polyhedron, giving rise to a linear N—Ni—N angle. The Ni—N bond length is 2.1158 (13) Å, and the Ni—O4 and Ni—O5 bond lengths are 2.0734 (11) and 2.0636 (12) Å, respectively. The N—Ni—O angles range from 82.22 (5) to 97.78 (5)° and the O—Ni—O angles from 89.94 (5) to 90.06 (5)°. Since the TEA ligands coordinate in their neutral form, charge compensation is required by two MHBA^−^ anions. They are in their benzoate form and are located in the outer coordination sphere, with the carboxyl­ate group tilted by 14.1 (2)° relative to the aromatic ring. The water mol­ecules are also non-coordinating.

## Supra­molecular features   

**Figure 2:**
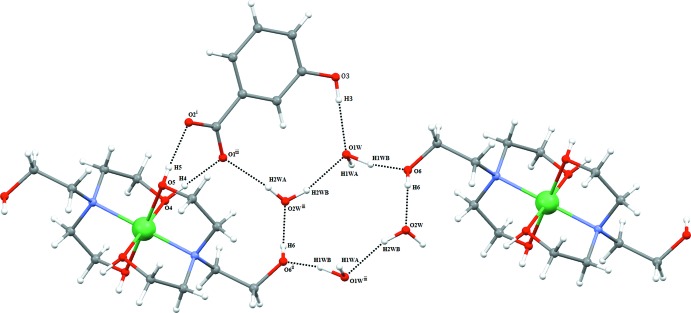
Different ring motifs generated by hydrogen bonds (shown as dashed lines). Symmetry codes refer to Table 1 ▸.

**Figure 3:**
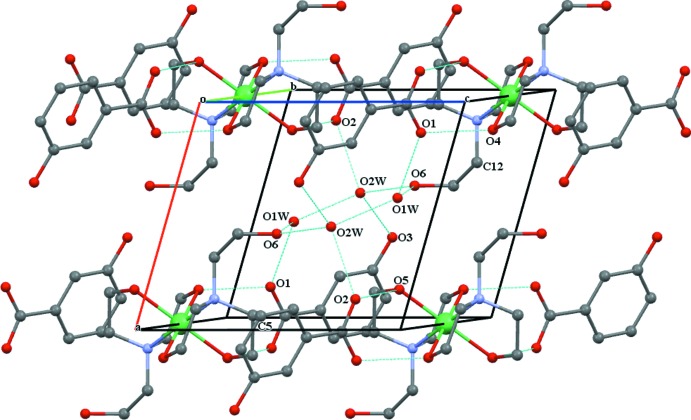
The packing of the mol­ecular entities in the crystal structure (shown as dashed lines). For clarity, H atoms have been omitted.

The supra­molecular structure features an intricate network of inter­molecular O—H⋯O hydrogen bonds (Table 1 ▸), including four cyclic motifs of different sizes. The MHBA^−^ anion is connected to the complex cation by a pair of rather strong hydrogen bonds [*D*⋯*A* = 2.579 (2) and 2.638 (2) Å, respectively] within a 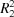
(8) motif (Etter, 1990 ▸) (Fig. 2 ▸). This ‘cation–anion’ hydrogen-bonded unit is further associated to the other moieties through formation of an 11-membered ring between the non-coordinating hydroxyl group O6 and water mol­ecule O2*W*. Three additional hydrogen bonds, O2*W*⋯O1, O3⋯O1*W* and O2*W*⋯O1*W*, lead to the same 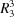
(11) graph-set motif, in each case with hydrogen bonds of medium strength (Table 1 ▸). The fourth cyclic motif has graph-set notation 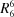
(12) and consists of a centrosymmetric 12-membered cycle between two unique water mol­ecules and the non-coordinating hydroxyl group O6 (Fig. 3 ▸). Together, the above-mentioned hydrogen-bonding inter­actions give rise to a two-dimensional supra­molecular structure extending parallel to (010).

## Database survey   

A survey of the Cambridge Structural Database (CSD) (Groom & Allen, 2014 ▸) showed that coordination complexes of TEA or MHBA with many metals including those of the *s*-, *d*-, *p*-, and *f*-block elements have been documented. 50 entries correspond to structures in which TEA mol­ecules are ligating, including 21 examples in a tetra­dentate mode (*e.g*. AKEXET, GEGTIV, IBOCOR, JOMDAS, LAKYAX, RUQSUR, SUTZIQ) and two polymeric structures (GOCVEZ, CUMSAE, CUMSAE01). The combination of tri- and tetra­dentate coordination modes is observed in five cases (MEVQIN, MEVQOT, EYIPAD, LAKYAX, MUCBIV). There is only one structure with TEA in a monodentate mode (KISMUW) and one with a bidentate mode (QAJDIP). The most frequently encountered tridentate coordination mode is also observed in the title compound and reported for 22 entries (*e.g.* ASUGEA, CABTEF, DAYPOJ, FOVKIL, ETOLNI, GUQXEV, IGALOR).

There are 40 entries for MHBA coordination complexes in the CSD. For 14 entries, the MHBA mol­ecules occupy a coordination sphere in the form of mixed-ligand complexes in monodentate coordination (*e.g*. GIMLEU, MEZFIG, NESFOH, SEZJOX), while bidentate coordination (*e.g.* MIQYIV, SISTAQ, WINFIJ, YIQQIZ) is found in twelve cases and a combination of the two modes only for entries CIVGOF and KIDBEE. Polymeric metal complex formation is reported for seven structures (CIWPIH, COSLAX, COSLIF, KIDBOO, COSKUQ, COSLEB, KIDBII). It should be noted that the hydroxyl group of the MHBA mol­ecule is involved in coordination neither in discrete nor in polymeric complexes. For five entries, MHBA mol­ecules are situated in the outer spheres (GANZAY, LAMMOD, MEWBOH, NIWJAF and WEJNIJ), as is the case in the title compound.

## Synthesis and crystallization   

To an aqueous solution (2.5 ml) of Ni(NO_3_)_2_ (0.091 g, 0.5 mmol) was slowly added an ethanol solution (5 ml) containing TEA (132 µl) and MHBA (0.138 g, 1 mmol) under constant stirring. A light-green crystalline product was obtained at room temperature by solvent evaporation after 25 days.

## Refinement   

Crystal data, data collection and structure refinement details are summarized in Table 2 ▸. C-bound hydrogen atoms were placed in calculated positions and refined in the riding-model approximation, with C—H = 0.93 and 0.97 Å for aromatic and methyl­ene hydrogen atoms, respectively, and with *U*_iso_(H) = 1.2*U*_eq_(C). O-bound hydrogen atoms were found from difference maps. Those attached to water mol­ecule O1*W* and to hy­droxy O atoms O4 and O5 were refined freely whereas those attached to water mol­ecule O2*W* and hy­droxy atoms O3 and O6 were refined with constrained O—H distances of 0.85 and 0.82 Å, respectively. For all O-bound hydrogen atoms, *U*_iso_(H) = 1.5*U*_eq_(O).

## Supplementary Material

Crystal structure: contains datablock(s) I. DOI: 10.1107/S2056989016005521/wm5282sup1.cif

Structure factors: contains datablock(s) I. DOI: 10.1107/S2056989016005521/wm5282Isup2.hkl

CCDC reference: 1471925

Additional supporting information:  crystallographic information; 3D view; checkCIF report

## Figures and Tables

**Table 1:** Hydrogen-bond geometry (Å, °)

*D*—H⋯*A*	*D*—H	H⋯*A*	*D*⋯*A*	*D*—H⋯*A*
O1*W*—H1*WA*⋯O2^i^	0.83 (3)	1.89 (3)	2.711 (2)	168 (3)
O1*W*—H1*WB*⋯O6^ii^	1.06 (5)	1.63 (3)	2.674 (2)	168 (4)
O2*W*—H2*WA*⋯O1^iii^	0.85	1.95	2.775 (2)	165
O2*W*—H2*WB*⋯O1*W* ^iii^	0.85	2.07	2.830 (2)	149
O3—H3⋯O1*W*	0.82	1.96	2.775 (2)	177
O4—H4⋯O1^iii^	0.87 (2)	1.72 (2)	2.579 (2)	169 (2)
O5—H5⋯O2^iii^	0.74 (3)	1.90 (3)	2.638 (2)	175 (3)
O6—H6⋯O2*W* ^iii^	0.82	1.93	2.728 (3)	165

Symmetry codes: (i) 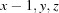; (ii) 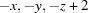; (iii) 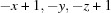.

**Table 2:** Experimental details

Crystal data	
Chemical formula	[Ni(C_6_H_15_NO_3_)_2_](C_7_H_5_O_3_)_2_·4H_2_O
*M*_r_	703.37
Crystal system, space group	Monoclinic, *P*2_1_/*n*
Temperature (K)	293
*a*, *b*, *c* (Å)	8.40515 (12), 21.4397 (3), 9.48944 (14)
β (°)	106.1835 (15)
*V* (Å^3^)	1642.27 (4)
*Z*	2
Radiation type	Cu *K*α
μ (mm^−1^)	1.50
Crystal size (mm)	0.32 × 0.14 × 0.12

Data collection	
Diffractometer	Agilent Xcalibur Ruby
Absorption correction	Multi-scan (*CrysAlis PRO*; Agilent, 2014 ▸)
*T*_min_, *T*_max_	0.912, 1.000
No. of measured, independent and observed [*I* > 2σ(*I*)] reflections	12555, 3399, 3066
*R*_int_	0.030
(sin θ/λ)_max_ (Å^−1^)	0.629

Refinement	
*R*[*F* ^2^ > 2σ(*F* ^2^)], *wR*(*F* ^2^), *S*	0.038, 0.115, 1.03
No. of reflections	3399
No. of parameters	226
No. of restraints	3
H-atom treatment	H atoms treated by a mixture of independent and constrained refinement
Δρ_max_, Δρ_min_ (e Å^−3^)	0.26, −0.44

Computer programs: *CrysAlis PRO* (Agilent, 2014 ▸), *OLEX2* (Dolomanov *et al.*, 2009 ▸) and *SHELXL2014* (Sheldrick, 2015 ▸).

## supplementary crystallographic information

### Crystal data

**Table:**

[Ni(C_6_H_15_NO_3_)_2_](C_7_H_5_O_3_)_2_·4H_2_O	*F*(000) = 748
*M**_r_* = 703.37	*D*_x_ = 1.422 Mg m^−^^3^
Monoclinic, *P*2_1_/*n*	Cu *K*α radiation, λ = 1.54184 Å
*a* = 8.40515 (12) Å	Cell parameters from 7605 reflections
*b* = 21.4397 (3) Å	θ = 4.1–75.8°
*c* = 9.48944 (14) Å	µ = 1.50 mm^−^^1^
β = 106.1835 (15)°	*T* = 293 K
*V* = 1642.27 (4) Å^3^	Prism, light-green
*Z* = 2	0.32 × 0.14 × 0.12 mm

### Data collection

**Table:**

Agilent Xcalibur Ruby diffractometer	3399 independent reflections
Radiation source: Enhance (Cu) X-ray Source	3066 reflections with *I* > 2σ(*I*)
Graphite monochromator	*R*_int_ = 0.030
Detector resolution: 10.2576 pixels mm^-1^	θ_max_ = 75.9°, θ_min_ = 4.1°
ω scans	*h* = −10→10
Absorption correction: multi-scan (*CrysAlis PRO*; Agilent, 2014)	*k* = −23→26
*T*_min_ = 0.912, *T*_max_ = 1.000	*l* = −11→7
12555 measured reflections	

### Refinement

**Table:**

Refinement on *F*^2^	Hydrogen site location: mixed
Least-squares matrix: full	H atoms treated by a mixture of independent and constrained refinement
*R*[*F*^2^ > 2σ(*F*^2^)] = 0.038	*w* = 1/[σ^2^(*F*_o_^2^) + (0.0717*P*)^2^ + 0.3396*P*] where *P* = (*F*_o_^2^ + 2*F*_c_^2^)/3
*wR*(*F*^2^) = 0.115	(Δ/σ)_max_ < 0.001
*S* = 1.03	Δρ_max_ = 0.26 e Å^−^^3^
3399 reflections	Δρ_min_ = −0.44 e Å^−^^3^
226 parameters	Extinction correction: *SHELXL2014* (Sheldrick, 2015), Fc^*^=kFc[1+0.001xFc^2^λ^3^/sin(2θ)]^-1/4^
3 restraints	Extinction coefficient: 0.0013 (3)

### Special details

**Table:**

Geometry. All esds (except the esd in the dihedral angle between two l.s. planes) are estimated using the full covariance matrix. The cell esds are taken into account individually in the estimation of esds in distances, angles and torsion angles; correlations between esds in cell parameters are only used when they are defined by crystal symmetry. An approximate (isotropic) treatment of cell esds is used for estimating esds involving l.s. planes.

### Fractional atomic coordinates and isotropic or equivalent isotropic displacement parameters (Å^2^)

**Table:**

	*x*	*y*	*z*	*U*_iso_*/*U*_eq_	
Ni1	0.5000	0.0000	0.5000	0.03393 (15)	
O4	0.64514 (15)	0.07781 (5)	0.49590 (13)	0.0419 (3)	
H4	0.658 (3)	0.0931 (8)	0.4149 (15)	0.063*	
O5	0.34872 (15)	0.02307 (7)	0.29528 (13)	0.0436 (3)	
O2	0.61445 (15)	−0.13123 (6)	0.82440 (16)	0.0516 (3)	
N1	0.38809 (17)	0.06100 (6)	0.61955 (15)	0.0393 (3)	
O1	0.34416 (15)	−0.11360 (6)	0.76086 (14)	0.0499 (3)	
C7	0.4724 (2)	−0.14099 (8)	0.83791 (18)	0.0404 (3)	
O3	0.1335 (2)	−0.23264 (8)	1.1285 (2)	0.0679 (4)	
H3	0.0720	−0.2045	1.0868	0.102*	
C3	0.2777 (2)	−0.23037 (8)	1.0902 (2)	0.0470 (4)	
C2	0.3022 (2)	−0.18851 (8)	0.98698 (19)	0.0417 (4)	
H2	0.2181	−0.1609	0.9419	0.050*	
C10	0.4602 (3)	0.04530 (10)	0.77728 (19)	0.0513 (4)	
H10A	0.3929	0.0133	0.8049	0.062*	
H10B	0.4571	0.0820	0.8362	0.062*	
C1	0.4506 (2)	−0.18721 (7)	0.94995 (18)	0.0391 (3)	
C9	0.6038 (3)	0.13094 (9)	0.5707 (3)	0.0576 (5)	
H9A	0.6147	0.1687	0.5179	0.069*	
H9B	0.6797	0.1337	0.6685	0.069*	
C6	0.5756 (3)	−0.22894 (9)	1.0159 (2)	0.0529 (5)	
H6A	0.6756	−0.2287	0.9919	0.063*	
C8	0.4288 (3)	0.12535 (9)	0.5813 (3)	0.0559 (5)	
H8A	0.4134	0.1541	0.6552	0.067*	
H8B	0.3526	0.1372	0.4880	0.067*	
C4	0.4027 (3)	−0.27183 (9)	1.1560 (2)	0.0576 (5)	
H4A	0.3879	−0.3001	1.2257	0.069*	
C11	0.6364 (3)	0.02244 (11)	0.8101 (2)	0.0569 (5)	
H11A	0.7093	0.0573	0.8081	0.068*	
H11B	0.6700	0.0043	0.9075	0.068*	
O6	0.1021 (3)	0.06378 (8)	0.7862 (2)	0.0809 (6)	
H6	0.0736	0.0271	0.7755	0.121*	
C5	0.5489 (3)	−0.27099 (10)	1.1178 (3)	0.0623 (5)	
H5A	0.6319	−0.2993	1.1614	0.075*	
C12	0.2050 (2)	0.05169 (10)	0.5721 (2)	0.0502 (4)	
H12A	0.1833	0.0079	0.5847	0.060*	
H12B	0.1663	0.0606	0.4679	0.060*	
C13	0.1006 (3)	0.08968 (11)	0.6485 (3)	0.0642 (5)	
H13A	0.1428	0.1320	0.6627	0.077*	
H13B	−0.0126	0.0914	0.5865	0.077*	
O2W	0.9448 (2)	0.06186 (9)	0.2035 (2)	0.0775 (5)	
H2WA	0.8479	0.0746	0.1994	0.116*	
H2WB	0.9679	0.0715	0.1246	0.116*	
O1W	−0.06671 (19)	−0.13462 (7)	0.99258 (17)	0.0543 (3)	
H1WA	−0.164 (4)	−0.1390 (14)	0.942 (3)	0.079 (9)*	
H1WB	−0.092 (6)	−0.103 (2)	1.070 (6)	0.164 (18)*	
H5	0.359 (3)	0.0545 (13)	0.266 (3)	0.061 (7)*	

### Atomic displacement parameters (Å^2^)

**Table:**

	*U*^11^	*U*^22^	*U*^33^	*U*^12^	*U*^13^	*U*^23^
Ni1	0.0372 (2)	0.0332 (2)	0.0320 (2)	−0.00273 (13)	0.01073 (15)	−0.00022 (13)
O4	0.0463 (6)	0.0390 (6)	0.0429 (6)	−0.0054 (5)	0.0164 (5)	0.0025 (5)
O5	0.0452 (6)	0.0453 (7)	0.0386 (6)	−0.0013 (5)	0.0085 (5)	0.0060 (5)
O2	0.0403 (6)	0.0553 (7)	0.0605 (8)	0.0006 (5)	0.0163 (6)	0.0172 (6)
N1	0.0426 (7)	0.0375 (7)	0.0391 (7)	−0.0020 (5)	0.0137 (6)	−0.0040 (5)
O1	0.0419 (6)	0.0581 (7)	0.0514 (7)	0.0046 (5)	0.0161 (5)	0.0212 (6)
C7	0.0417 (8)	0.0388 (8)	0.0408 (8)	−0.0003 (6)	0.0115 (6)	0.0049 (6)
O3	0.0679 (10)	0.0702 (10)	0.0772 (10)	0.0026 (7)	0.0396 (8)	0.0252 (8)
C3	0.0547 (10)	0.0437 (9)	0.0450 (9)	−0.0046 (7)	0.0179 (8)	0.0048 (7)
C2	0.0446 (9)	0.0387 (8)	0.0408 (8)	0.0010 (6)	0.0100 (7)	0.0068 (6)
C10	0.0624 (11)	0.0543 (10)	0.0376 (9)	−0.0011 (8)	0.0148 (8)	−0.0101 (7)
C1	0.0427 (8)	0.0358 (8)	0.0375 (8)	−0.0021 (6)	0.0088 (6)	0.0033 (6)
C9	0.0719 (13)	0.0399 (9)	0.0674 (12)	−0.0155 (8)	0.0303 (10)	−0.0105 (8)
C6	0.0481 (10)	0.0477 (10)	0.0625 (12)	0.0070 (8)	0.0145 (8)	0.0125 (8)
C8	0.0698 (13)	0.0356 (9)	0.0706 (12)	0.0017 (8)	0.0331 (10)	−0.0030 (8)
C4	0.0707 (13)	0.0491 (10)	0.0519 (11)	−0.0012 (9)	0.0154 (9)	0.0189 (8)
C11	0.0608 (12)	0.0601 (11)	0.0409 (9)	−0.0055 (9)	−0.0005 (8)	−0.0077 (8)
O6	0.1092 (15)	0.0688 (10)	0.0874 (12)	−0.0059 (10)	0.0650 (12)	−0.0136 (9)
C5	0.0629 (12)	0.0523 (11)	0.0677 (13)	0.0123 (9)	0.0115 (10)	0.0233 (10)
C12	0.0419 (9)	0.0595 (11)	0.0515 (10)	0.0006 (8)	0.0169 (8)	−0.0084 (8)
C13	0.0583 (12)	0.0652 (13)	0.0775 (14)	0.0078 (10)	0.0331 (11)	−0.0063 (11)
O2W	0.0663 (10)	0.0802 (11)	0.0964 (14)	0.0154 (9)	0.0401 (10)	0.0171 (10)
O1W	0.0444 (7)	0.0649 (9)	0.0559 (8)	0.0029 (6)	0.0179 (6)	−0.0039 (6)

### Geometric parameters (Å, º)

**Table:**

Ni1—O4^i^	2.0735 (11)	C1—C6	1.389 (2)
Ni1—O4	2.0734 (11)	C9—H9A	0.9700
Ni1—O5	2.0636 (12)	C9—H9B	0.9700
Ni1—O5^i^	2.0636 (12)	C9—C8	1.507 (3)
Ni1—N1^i^	2.1158 (13)	C6—H6A	0.9300
Ni1—N1	2.1158 (13)	C6—C5	1.385 (3)
O4—H4	0.869 (9)	C8—H8A	0.9700
O4—C9	1.435 (2)	C8—H8B	0.9700
O5—C11^i^	1.427 (2)	C4—H4A	0.9300
O5—H5	0.74 (3)	C4—C5	1.375 (3)
O2—C7	1.253 (2)	C11—O5^i^	1.427 (2)
N1—C10	1.488 (2)	C11—H11A	0.9700
N1—C8	1.491 (2)	C11—H11B	0.9700
N1—C12	1.491 (2)	O6—H6	0.8200
O1—C7	1.266 (2)	O6—C13	1.417 (3)
C7—C1	1.501 (2)	C5—H5A	0.9300
O3—H3	0.8200	C12—H12A	0.9700
O3—C3	1.359 (2)	C12—H12B	0.9700
C3—C2	1.386 (2)	C12—C13	1.521 (3)
C3—C4	1.385 (3)	C13—H13A	0.9700
C2—H2	0.9300	C13—H13B	0.9700
C2—C1	1.387 (2)	O2W—H2WA	0.8498
C10—H10A	0.9700	O2W—H2WB	0.8500
C10—H10B	0.9700	O1W—H1WA	0.83 (3)
C10—C11	1.508 (3)	O1W—H1WB	1.07 (5)
			
O4—Ni1—O4^i^	180.0	C2—C1—C6	119.57 (16)
O4^i^—Ni1—N1^i^	82.22 (5)	C6—C1—C7	121.18 (16)
O4—Ni1—N1^i^	97.78 (5)	O4—C9—H9A	109.6
O4—Ni1—N1	82.22 (5)	O4—C9—H9B	109.6
O4^i^—Ni1—N1	97.78 (5)	O4—C9—C8	110.20 (15)
O5—Ni1—O4^i^	89.95 (5)	H9A—C9—H9B	108.1
O5^i^—Ni1—O4	89.94 (5)	C8—C9—H9A	109.6
O5—Ni1—O4	90.05 (5)	C8—C9—H9B	109.6
O5^i^—Ni1—O4^i^	90.06 (5)	C1—C6—H6A	120.5
O5^i^—Ni1—O5	180.0	C5—C6—C1	118.99 (19)
O5—Ni1—N1	96.16 (5)	C5—C6—H6A	120.5
O5—Ni1—N1^i^	83.84 (5)	N1—C8—C9	112.60 (16)
O5^i^—Ni1—N1^i^	96.16 (5)	N1—C8—H8A	109.1
O5^i^—Ni1—N1	83.84 (5)	N1—C8—H8B	109.1
N1^i^—Ni1—N1	180.0	C9—C8—H8A	109.1
Ni1—O4—H4	122.6 (12)	C9—C8—H8B	109.1
C9—O4—Ni1	113.85 (10)	H8A—C8—H8B	107.8
C9—O4—H4	104.1 (12)	C3—C4—H4A	120.2
Ni1—O5—H5	118 (2)	C5—C4—C3	119.62 (17)
C11^i^—O5—Ni1	110.15 (11)	C5—C4—H4A	120.2
C11^i^—O5—H5	108 (2)	O5^i^—C11—C10	110.50 (15)
C10—N1—Ni1	106.39 (11)	O5^i^—C11—H11A	109.5
C10—N1—C8	113.44 (15)	O5^i^—C11—H11B	109.5
C10—N1—C12	111.71 (14)	C10—C11—H11A	109.5
C8—N1—Ni1	105.96 (11)	C10—C11—H11B	109.5
C8—N1—C12	109.74 (15)	H11A—C11—H11B	108.1
C12—N1—Ni1	109.32 (10)	C13—O6—H6	109.5
O2—C7—O1	123.06 (15)	C6—C5—H5A	119.2
O2—C7—C1	119.38 (15)	C4—C5—C6	121.53 (18)
O1—C7—C1	117.56 (14)	C4—C5—H5A	119.2
C3—O3—H3	109.5	N1—C12—H12A	107.8
O3—C3—C2	122.12 (17)	N1—C12—H12B	107.8
O3—C3—C4	118.51 (17)	N1—C12—C13	117.92 (17)
C4—C3—C2	119.37 (18)	H12A—C12—H12B	107.2
C3—C2—H2	119.5	C13—C12—H12A	107.8
C3—C2—C1	120.92 (16)	C13—C12—H12B	107.8
C1—C2—H2	119.5	O6—C13—C12	111.83 (19)
N1—C10—H10A	109.1	O6—C13—H13A	109.2
N1—C10—H10B	109.1	O6—C13—H13B	109.2
N1—C10—C11	112.46 (15)	C12—C13—H13A	109.2
H10A—C10—H10B	107.8	C12—C13—H13B	109.2
C11—C10—H10A	109.1	H13A—C13—H13B	107.9
C11—C10—H10B	109.1	H2WA—O2W—H2WB	109.4
C2—C1—C7	119.25 (14)	H1WA—O1W—H1WB	97 (3)
			
Ni1—O4—C9—C8	−22.1 (2)	C3—C2—C1—C7	−179.82 (16)
Ni1—N1—C10—C11	31.50 (18)	C3—C2—C1—C6	0.8 (3)
Ni1—N1—C8—C9	−40.0 (2)	C3—C4—C5—C6	0.9 (4)
Ni1—N1—C12—C13	177.98 (15)	C2—C3—C4—C5	−0.2 (3)
O4—C9—C8—N1	42.1 (2)	C2—C1—C6—C5	−0.1 (3)
O2—C7—C1—C2	166.38 (16)	C10—N1—C8—C9	76.4 (2)
O2—C7—C1—C6	−14.2 (3)	C10—N1—C12—C13	60.5 (2)
N1—C10—C11—O5^i^	−46.7 (2)	C1—C6—C5—C4	−0.7 (4)
N1—C12—C13—O6	−79.9 (2)	C8—N1—C10—C11	−84.60 (19)
O1—C7—C1—C2	−13.7 (2)	C8—N1—C12—C13	−66.2 (2)
O1—C7—C1—C6	165.70 (18)	C4—C3—C2—C1	−0.6 (3)
C7—C1—C6—C5	−179.51 (19)	C12—N1—C10—C11	150.73 (16)
O3—C3—C2—C1	180.00 (18)	C12—N1—C8—C9	−157.89 (17)
O3—C3—C4—C5	179.2 (2)		

Symmetry code: (i) −*x*+1, −*y*, −*z*+1.

### Hydrogen-bond geometry (Å, º)

**Table:**

*D*—H···*A*	*D*—H	H···*A*	*D*···*A*	*D*—H···*A*
O1*W*—H1*WA*···O2^ii^	0.83 (3)	1.89 (3)	2.711 (2)	168 (3)
O1*W*—H1*WB*···O6^iii^	1.06 (5)	1.63 (3)	2.674 (2)	168 (4)
O2*W*—H2*WA*···O1^i^	0.85	1.95	2.775 (2)	165
O2*W*—H2*WB*···O1*W*^i^	0.85	2.07	2.830 (2)	149
O3—H3···O1*W*	0.82	1.96	2.775 (2)	177
O4—H4···O1^i^	0.87 (2)	1.72 (2)	2.579 (2)	169 (2)
O5—H5···O2^i^	0.74 (3)	1.90 (3)	2.638 (2)	175 (3)
O6—H6···O2*W*^i^	0.82	1.93	2.728 (3)	165

Symmetry codes: (i) −*x*+1, −*y*, −*z*+1; (ii) *x*−1, *y*, *z*; (iii) −*x*, −*y*, −*z*+2.
